# Current Progress in Pharmacogenetics of Second-Line Antidiabetic Medications: Towards Precision Medicine for Type 2 Diabetes

**DOI:** 10.3390/jcm8030393

**Published:** 2019-03-21

**Authors:** Chan Uk Heo, Chang-Ik Choi

**Affiliations:** College of Pharmacy, Dongguk University-Seoul, Goyang 10326, Korea; dgucpt001@gmail.com

**Keywords:** pharmacogenetics, precision medicine, personalized medicine, type 2 diabetes, second-line antidiabetic medications, DPP-4 inhibitors, GLP-1 receptor agonists, SGLT2 inhibitors

## Abstract

Precision medicine is a scientific and medical practice for personalized therapy based on patients’ individual genetic, environmental, and lifestyle characteristics. Pharmacogenetics and pharmacogenomics are also rapidly developing and expanding as a key element of precision medicine, in which the association between individual genetic variabilities and drug disposition and therapeutic responses are investigated. Type 2 diabetes (T2D) is a chronic metabolic disorder characterized by hyperglycemia mainly associated with insulin resistance, with the risk of clinically important cardiovascular, neurological, and renal complications. The latest consensus report from the American Diabetes Association and European Association for the Study of Diabetes (ADA-EASD) on the management of T2D recommends preferential use of glucagon-like peptide-1 (GLP-1) receptor agonists, sodium-glucose cotransporter-2 (SGLT2) inhibitors, and some dipeptidyl peptidase-4 (DPP-4) inhibitors after initial metformin monotherapy for diabetic patients with established atherosclerotic cardiovascular or chronic kidney disease, and with risk of hypoglycemia or body weight-related problems. In this review article, we summarized current progress on pharmacogenetics of newer second-line antidiabetic medications in clinical practices and discussed their therapeutic implications for precision medicine in T2D management. Several biomarkers associated with drug responses have been identified from extensive clinical pharmacogenetic studies, and functional variations in these genes have been shown to significantly affect drug-related glycemic control, adverse reactions, and risk of diabetic complications. More comprehensive pharmacogenetic research in various clinical settings will clarify the therapeutic implications of these genes, which may be useful tools for precision medicine in the treatment and prevention of T2D and its complications.

## 1. Introduction

Precision medicine is a scientific and medical practice in which patients are provided tailored treatments based on their individual characteristics including genetic, environmental, and lifestyle factors [1]. The National Research Council recommends that this term should be clearly distinguished from personalized medicine, because the word “personalized” can be misinterpreted to imply that treatments or preventions are being developed uniquely for individual patients [2]. However in many cases, people use these terms interchangeably. Governments and regulatory authorities in developed countries have been implementing new policies for the realization of precision medicine [3,4].

As a part of precision medicine, the field of pharmacogenetics and pharmacogenomics is also rapidly developing and expanding. Starting with the Human Genome Project in 1990, many follow-up projects have been conducted to identify the genes and genetic variations involved in disease development [5]. These genetic variations have been found to affect disease occurrence, progression, or recurrence, type or dose of drug most likely to be effective, nature and extent of therapeutic responses, and risk of adverse drug reactions [1]. More than 170 genes are reportedly involved in drug disposition, and more than half have functional genetic polymorphisms [6], leading to alterations in drug pharmacokinetics (PK) and/or pharmacodynamics (PD). The U.S Food and Drug Administration (FDA) regularly updates and provides the latest list of pharmacogenomic biomarkers in drug labeling to ensure the effective and safe use of drugs [7], with more than one-third (38%) related to the therapeutic efficacy of anticancer drugs.

Type 2 diabetes (T2D) is a chronic metabolic disorder characterized by hyperglycemia resulting from defects in insulin secretion, insulin action, or both. Because several clinically important cardiovascular, neurological, and renal complications can lead to increased morbidity and mortality in diabetic patients, the primary goal of T2D treatment is to prevent or delay complications via strict management of blood glucose and cardiovascular risk factors, as well as self-care activities [8,9]. Among the 12 different classes of drugs available for the treatment of T2D (Table 1), metformin is widely recommended as the first-line glucose-lowering agent if there is no contraindication, due to its high efficacy, low-cost, and minimal drug-induced toxicity such as hypoglycemia [10]. Therefore, pharmacogenetic studies of antidiabetic drugs have also been primarily conducted on identification of candidate genes involved in metformin PK and/or therapeutic responses. A number of studies have demonstrated that the major clinically important genes related to metformin treatment are responsible for the expression of solute carrier (SLC)-type transporters, such as organic cation transporter (OCT) 1 (*SLC22A1*), OCT2 (*SLC22A2*), multidrug and toxin extrusion (MATE) 1 (*SLC47A1*), and MATE2-K (*SLC47A2*) [11,12,13]. These transporters are mainly located in human hepatocytes and renal proximal tubules [14] and are responsible for the plasma exposure of metformin, leading to its clinical efficacy on blood glucose, glycated hemoglobin (HbA1c), and insulin sensitivity.

**Figure 1:**
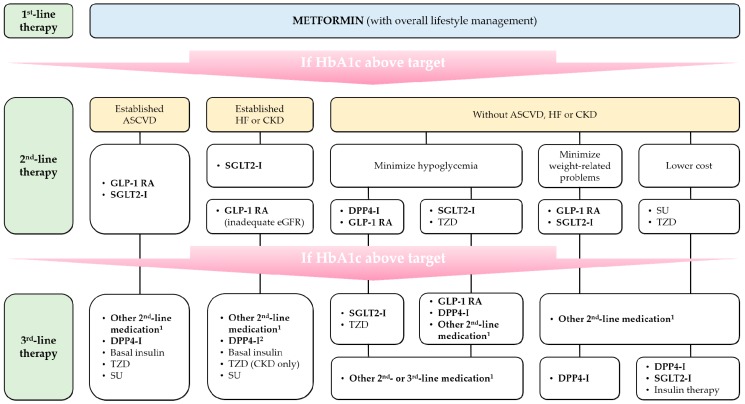
Schematic overview of management of T2D by the 2018 ADA-EASD consensus report (Adapted from Ref. [10], by permission of Springer Nature.). ^1^ drug classes not selected in previous step(s); ^2^ saxagliptin is excluded. ASCVD, atherosclerotic cardiovascular disease; CKD, chronic kidney disease; DPP4-I, DPP-4 inhibitor; eGFR, estimated glomerular filtration rate; GLP-1 RA, GLP-1 receptor agonist; HF, heart failure; HbA1c, glycated hemoglobin; SGLT2-I, SGLT2 inhibitor; SU, sulfonylurea; TZD, thiazolidinedione.

If metformin monotherapy fails to achieve adequate therapeutic effects or causes inappropriate adverse drug reactions, other medications can be added to or replace metformin. According to a recent consensus report on the management of hyperglycemia in T2D by the American Diabetes Association and European Association for the Study of Diabetes (ADA-EASD) [10], sulfonylureas, thiazolidinediones, dipeptidyl peptidase-4 (DPP-4) inhibitors, glucagon-like peptide-1 (GLP-1) receptor agonists, and sodium-glucose cotransporter-2 (SGLT2) inhibitors are considered reasonable options for second-line glucose-lowering agents. However, despite the economic advantages, the use of sulfonylureas and thiazolidinediones is gradually diminishing due to the risk of drug-induced hypoglycemia and weight gain and uncertain cardiovascular safety. Instead, the ADA-EASD recommends the preferential use of GLP-1 receptor agonists, SGLT2 inhibitors, and some DPP-4 inhibitors after initial metformin monotherapy for T2D patients with established atherosclerotic cardiovascular disease (ASCVD) or chronic kidney disease (CKD), and patients who need to minimize hypoglycemia and/or body weight-related problems (Figure 1) [10].

Based on this paradigm shift to patient-centered T2D treatment strategy, we summarized and reviewed current progress in pharmacogenetics of these new-generation second-line antidiabetic agents in clinical practices and discussed their therapeutic implications for precision medicine in the management of T2D and its complications.

## 2. DPP-4 Inhibitors

The concept of “incretin effect” was first recognized in the 1960s, by the observation that enteral glucose administration provided a more potent insulinotropic stimulus in pancreas compared with isoglycemic intravenous infusion [16]. Until now, two endogenous incretin hormones—glucose-dependent insulinotropic polypeptide (GIP) and GLP-1—were identified and their physiological roles in human body have been demonstrated such as glucose-dependent stimulation of insulin and suppression of glucagon releases [17,18], insulin biosynthesis and gene transcription activation [19], enhancement of β-cell growth and regeneration and apoptosis inhibition [20,21,22], gastric emptying inhibition [23], and increased glucose uptake and glycogen synthesis in muscles, liver, and adipose tissues [24].

Despite clinically significant roles of incretin hormones in glucose homeostasis, they have a critical drawback associated with extremely short plasma half-lives (<2 min). Once secreted into the bloodstream, they are immediately metabolized by the proteolytic enzyme DPP-4 into inactive metabolite [25]. Therefore, the development of specific protease inhibitors that prevent rapid inactivation of plasma incretin hormones has been considered a novel therapeutic strategy for the management of T2D. Since the first DPP-4 inhibitor—sitagliptin—was released on the market with the approval of the U.S FDA in 2006 [26], many pharmaceutical companies have developed new DPP-4 inhibitors and a total of 12 products (alogliptin, anagliptin, evogliptin, gemigliptin, gosogliptin, linagliptin, omarigliptin, saxagliptin, sitagliptin, teneligliptin, trelagliptin, and vildagliptin) are currently available worldwide [27].

DPP-4 inhibitors are used as monotherapy or in various combination regimens with other antidiabetic agents due to their good efficacy, tolerability, and favorable safety profiles [28]. Because of the relative weight-neutral effect of DPP-4 inhibitors [29] and a recent clinical outcomes trial report on increased risk of hospitalization for heart failure attributable to saxagliptin use (the risk of “class effect”) [30], the ADA-EASD recommends the preferred consideration of GLP-1 receptor agonists over DPP-4 inhibitors in T2D patients with ASCVD or weight-related problems. Nevertheless, DPP-4 inhibitors are still attractive second-line medications as they can be orally available, leading to increased patient compliance in T2D patients compared with GLP-1 receptor agonists which are administered by subcutaneous injection [31]. Genes associated with therapeutic responses to DPP-4 inhibitors were summarized in Table 2.

### 2.1. Adenosine Triphosphate-Binding Cassette Subfamily B Member 1 (ABCB1)

The first study on pharmacogenetics of DPP-4 inhibitors was conducted by Aquilante et al. [41], in which they investigated the effect of genetic polymorphisms in *ABCB1* (a gene encoding efflux transporter P-glycoprotein (P-gp)) and atorvastatin, which is both a substrate and an inhibitor of P-gp, on sitagliptin PK in healthy volunteers. A total of 29 subjects (16 males and 13 females) were genotyped for three *ABCB1* single-nucleotide polymorphisms (SNPs)—rs1128503 (c.1236C > T), rs2032582 (c.2677G > T/A), and rs1045642 (c.3435C > T)—and classified into three different groups according to *ABCB1* (1236/2677/3435) diplotype. Although modest alterations in the maximum plasma concentration (C_max_), area under the plasma concentration–time curve (AUC), renal clearance (CL_R_), and fraction excreted unchanged in the urine (f_e_) of sitagliptin were observed in *ABCB1* TTT/TTT diplotype subjects (*n* = 10) and, compared with the two other diplotype groups (*n* = 9 for CGC/CGC; *n* = 10 for CGC/TTT), all differences did not reach statistical significance. Concomitant administration of atorvastatin also did not affect sitagliptin PK among three diplotype groups. Despite previous in vitro and clinical research data that sitagliptin is a substrate for P-gp [42,43], the influence of *ABCB1* genetic polymorphisms on sitagliptin disposition was concluded to be negligible.

### 2.2. DPP4

The effects of *DPP4* genetic polymorphisms on the efficacy of DPP-4 inhibitors were first reported with vildagliptin [44]. Of the 14 SNPs discovered in this study, six (rs13015258 A > C; rs17848920 G > A; rs10930040 C > T; rs2302873 G > A; rs17848910 C > T; rs2268891 T > C) were selected and their influence on vildagliptin responses were evaluated in 48 T2D patients. However, no genetic variant strongly affected the clinical efficacy of vildagliptin.

More recently, the effects of genetic variants in *DPP4* on sitagliptin treatment were also evaluated [32]. A total of 27 T2D patients with hypertension and 38 healthy controls participated in a randomized, double-blind, placebo-controlled crossover study. Patients were allowed to take their existing antihyperglycemic and antihypertensive medications throughout the study period. All subjects were randomly administered repeated doses of 100 mg sitagliptin daily for four or seven days, a single dose of 200 mg sitagliptin or matching placebo, and relationship between *DPP4* genotypes and DPP-4 activity during placebo or sitagliptin treatment was evaluated. The authors found that two genetic variants (rs2909451 C > T; rs759717 G > C) were associated with increased DPP-4 activity during sitagliptin treatment. Furthermore, in multivariable analyses, rs2909451 TT genotype was considered one of the significant factors on DPP-4 activity during sitagliptin treatment.

### 2.3. GLP-1 Receptor Gene (GLP1R)

As the role of GLP-1 receptors in the glucose-lowering mechanisms of DPP-4 inhibitors was recently reported [45], the hypothesis that alterations in the structure or functional affinity of GLP-1 receptors can influence therapeutic responses to DPP-4 inhibitors has emerged. In an exploratory study of 246 Korean T2D patients with DPP-4 inhibitor treatment for at least 24 weeks, the effects of the *GLP1R* rs3765467 G > A genetic variant on DPP-4 inhibitor responses were investigated [33]. More than 90% of patients received vildagliptin (*n* = 151; 61.4% of total) or sitagliptin (*n* = 82; 33.3% of total). Reduced HbA1c levels and DPP-4 inhibitor response rates in patients carrying the rs3765467 GA/AA genotype (*n* = 77) were significantly greater than those in patients with the GG genotype (*n* = 169; *p* = 0.022 and *p* = 0.018, respectively). This tendency was particularly pronounced in stratified patients with higher baseline HbA1c (>8.0%) (*p* = 0.005). Results from multivariate logistic analyses have also supported the fact that patients with rs3765467 A-allele (GA/AA genotype) showed better treatment responses to DPP-4 inhibitors than patients with the GG genotype after other confounding factors were adjusted.

Another study by Javorský et al. [34] also demonstrated that a missense variant in *GLP1R* (rs6923761; p.Gly168Ser) is associated with reduced responses to DPP-4 inhibitor treatment. Reduction in HbA1c levels (%) in patients with Gly/Gly (*n* = 50; −0.90 ± 0.14%) and Gly/Ser (*n* = 72; −0.73 ± 0.12%) genotypes were greater than that in Ser/Ser genotype patients (*n* = 18; −0.12 ± 0.23%), reaching statistical significance in all genetic study models (additive, codominant, and recessive). The influence of the GIP receptor gene (*GIPR*) rs10423928 variant was also evaluated in this study, but no significant relationship was observed.

### 2.4. Transcription Factor 7-Like 2 (TCF7L2)

Genetic polymorphisms in *TCF7L2* affect the therapeutic response to insulin secretagogues (e.g., sulfonylureas) and increase the risk of T2D, with the underlying mechanism of impaired insulinotropic action of incretin hormones [46,47,48,49]. Zimdahl et al. [35] investigated the influence of *TCF7L2* risk variants (rs7903146 C > T) on the clinical efficacy and safety of linagliptin in patients taking linagliptin as monotherapy or in combination with other medications for T2D treatment. They were divided into three groups based on *TCF7L2* genotype (*n* = 356 for CC genotype; *n* = 264 for CT; *n* = 73 for TT). Impaired decrease in HbA1c level (%) with 24-week linagliptin therapy was observed in patients with the rs7903146 TT genotype (−0.57%) compared with other genotype groups (−0.82% for CC; −0.77% for CT). The difference in response to linagliptin between TT and CC genotype carriers reached statistical significance (*p* = 0.0182). The effects of *TCF7L2* rs7903146 genotype on changes in 2-h postprandial plasma glucose from baseline (2-h PG) were further studied in a limited number of patients. Similar to the results of HbA1c, patients with TT genotype showed the lowest mean decrease in 2-h PG (1.65 mmol/L), followed by the CT genotype (2.55 mmol/L) and CC genotype (2.78 mmol/L) groups.

### 2.5. Patatin-Like Phospholipase 3 (PNPLA3)

Non-alcoholic fatty liver disease (NAFLD), characterized by abnormal fat accumulation in hepatocytes, is closely related to insulin resistance and metabolic syndrome [50]. The rs738409 C > G SNP (p.Ile148Met) in *PNPLA3* was found to be associated with impaired hydrolysis of emulsified triglycerides, leading to increased free fatty acids and triglyceride levels and insulin resistance in the liver [51,52]. Kan et al. [36] investigated the effects of alogliptin treatment on liver function and glucose metabolism in NAFLD patients with T2D, carrying different *PNPLA3* rs738409 C > G genotypes. A total of 41 study subjects (17 males and 24 females) with a mean age of 60.5 years were treated with 25 mg alogliptin daily for approximately 33 months, and comparative analyses of their clinical and laboratory characteristics before and after drug administration were performed. After alogliptin treatment, a more positive correlation between improvements in HbA1c levels and changes in liver transaminase levels were observed in patients with G-allele (CG or GG genotype; *n* = 28). In a further investigation of 27 patients who experienced weight loss during the study periods, patients with the CG+GG genotype (*n* = 18) showed significant improvements in total cholesterol, triglyceride (TG) and hyaluronic acid compared with CC-genotype carriers (*n* = 9). These findings indicate that alogliptin (and other DPP-4 inhibitors) can affect liver function in NAFLD patients proportionally with the extent of improvement in their HbA1c levels, and these effects are influenced by rs738409 genetic polymorphism in *PNPLA3*.

### 2.6. Cyclin-Dependent Kinase 5 Regulatory Subunit Associated Protein 1-Like 1 (CDKAL1)

Previous genome-wide association studies (GWAS) have showed the relationship between *CDKAL1* gene and T2D susceptibility, and two common variants in *CDKAL1*—rs7754840 G > C and rs7756992 A > G—were associated with increased risk of T2D in Japanese [53,54]. Osada et al. [37] performed a retrospective, historical cohort study on the association between these common variants and therapeutic responses to antidiabetic drugs in T2D patients. Unlike other medications, HbA1c reduction in patients who received a DPP-4 inhibitor (*n* = 512, regardless of concomitant use of other antidiabetic agents) showed significant differences according to *CDKAL1* rs7754840 G > C (−0.4% in GG genotype; −0.5% in GC; −0.8% in CC; *p* = 0.02) and rs7756992 A > G (−0.4% in AA genotype; −0.5% in AG; −0.8% in GG; *p* = 0.01) genotypes. Further sensitivity analyses have shown more obvious distinctions in HbA1c reduction between different *CDKAL1* genotypes when the data were limited to patients who continued use of DPP-4 inhibitor throughout study period, or to patients who initiated combination drug therapy including DPP-4 inhibitor. After other covariates, including age, sex, body mass index (BMI), diabetes duration, HbA1c, and the number of concomitant antidiabetic drugs, were adjusted in a multivariate linear regression analysis, reductions of HbA1c in patients with DPP-4 inhibitor treatment showed significant associations with *CDKAL1* rs7754840 and rs7756992 risk alleles until the treatment was maintained after 12 months (*p*-value at 12 months = 0.01 for rs7754840; 0.002 for rs7756992).

### 2.7. Potassium Channels (KCN) Gene Family

The effects of genetic polymorphisms in genes related to the response to DPP-4 inhibitors (*DPP4*, wolframin endoplasmic reticulum transmembrane glycoprotein (*WFS1*), and potassium voltage-gated channel subfamily J member 11 (*KCNJ11*)) were determined in 662 T2D patients treated with sitagliptin, vildagliptin, or linagliptin (*n* = 331), or with other antidiabetic medications (control group, *n* = 331) [38]. Of nine selected SNPs from three genes, *WFS1* rs734312 A > G (odds ratio (OR) = 1.697; X^2^ = 5.479; *p* = 0.0192; Hardy–Weinberg equilibrium (HWE) = 0.7759) and *KCNJ11* rs2285676 C > T (OR = 1.479; X^2^ = 4.559; *p* = 0.0327; HWE = 0.9596) exhibited a strong association with DPP-4 inhibitor therapeutic efficacy. Since *KCNJ11* is known to regulate incretin signaling pathway of insulin secretion from pancreatic β-cells [55,56], the results of predictive modeling analysis consequently indicated that the *KCNJ11* rs2285676 variant is a genetic predictor of DPP-4 inhibitor treatment responses. Another potassium voltage-gated channel related to QT interval (KQT)-like subfamily member 1 gene (*KCNQ1*) is also responsible for insulin release from pancreatic β-cells and GLP-1 storage and secretion from intestinal L-cells [57,58]. Gotthardová et al. [39] demonstrated the association between genetic polymorphisms in *KCNQ1* and therapeutic responses to DPP-4 inhibitor treatment in 137 T2D patients receiving a daily dose of 100 mg sitagliptin (*n* = 90) or vildagliptin (*n* = 47). *KCNQ1* rs163184 T > G and rs151290 C > A variants were selected for the study based on previous reports demonstrating the roles of these SNPs on T2D susceptibility and secretion of incretin hormones and insulin [59,60,61,62]. In additive genetic model analyses, the *KCNQ1* rs163184 variant exhibited a significant association with diminished DPP-4 inhibitor responses (*β* = −0.30; *p* = 0.022). Further analyses in a dominant genetic model showed significantly lower reduction in HbA1c levels after DPP-4 inhibitor treatment was observed in patients carrying rs163184 G-allele (*p* = 0.037). The mean difference in HbA1c reduction between rs163184 TT and GG genotype patients was 0.6% (*p* = 0.021).

### 2.8. Protein Kinase D1 (PRKD1)

Liao et al. [40] utilized a genome-wide association study (GWAS)-based approach to find candidate genes affecting therapeutic responses to DPP-4 inhibitors in 171 Taiwanase patients with T2D receiving sitagliptin (*n* = 114), saxagliptin (*n* = 22), vildagliptin (*n* = 23), or linagliptin (*n* = 12) therapy for at least 60 days. Before analyses, they were further classified into two groups based on their sensitivity to antidiabetic therapy (88 resistant responders and 83 sensitive responders). In a preliminary GWAS, 45 SNPs were found to be associated with DPP-4 inhibitor responses, with the strongest association observed in rs57803087 variant located within the fourth intron of *PRKD1* gene (*p* = 3.2 × 10^−6^). A subsequent replication study was performed with the six most significant SNPs (*PRKD1* rs57803087—contactin 3 (*CNTN3*) rs10511037 and rs62266510, apoptosis signal-regulating kinase 1 (*ASK1*) rs7755097 and rs9376211, and *LOC105377923* rs4946688 and rs1948999)—and only *PRKD1* rs57803087 still exhibited a significant association with DPP-4 inhibitor therapeutic efficacy. PRKD1 is a serine/threonine kinase involving G protein-coupled receptor 40 mediated insulin secretion from pancreatic β-cells [63], suggesting that the genes associated with β-cell functions have potentials to be beneficial to the clinical efficacy of DPP-4 inhibitors.

## 3. GLP-1 Receptor Agonists

As another therapeutic strategy to overcome DPP-4-mediated rapid inactivation of endogenous incretin hormones, efforts have been made to identify new peptides that have a similar structure to incretin hormones and are not affected by DPP-4. In particular, many researchers and pharmaceutical companies have paid attention to the structural characteristics of GLP-1, because GLP-1, but not GIP, has relatively preserved insulinotropic activity in diabetic patients [64].

The first GLP-1 receptor agonist exenatide (a synthetic product of exendin-4) was originally derived from the saliva of *Heloderma suspectum* [65], with 53% homology with human GLP-1. Its antidiabetic potentials have been evaluated in several preclinical experiments [66,67,68] and clinical trials [69,70,71,72,73,74,75,76], and the U.S FDA approved exenatide as a new class of antihyperglycemic agent in 2005 [77].

To date, a total of six GLP-1 receptor agonists—albiglutide, dulaglutide, exenatide (including extended release (ER) formulation), liraglutide, lixisenatide, and semaglutide—have been developed and entered the market. Newer medications except lixisenatide have over 90% amino acid sequence identity to native human GLP-1, leading to a lower risk of antibody formation to these peptide drugs [78,79,80]. Dosing intervals affecting patient compliance have been also improved, from twice-daily (exenatide) to once-daily (liraglutide and lixisenatide) or once-weekly (albiglutide, dulaglutide, exenatide ER, and semaglutide) [78]. Recent studies suggest that GLP-1 receptor agonists can replace rapid-acting insulin in basal insulin therapy, with cost-saving benefits, effective glycemic control, and reduced risk of weight gain and hypoglycemia in patients with T2D uncontrolled on conventional basal insulin regimen [81,82,83,84,85]. Despite the inconvenience of administration route, GLP-1 receptor agonists are effective second-line medications after initial metformin treatment due to their additional actions including body weight reduction [86], lower risk for hypoglycemia [87], and improved cardiovascular outcomes [88]. Because it was already known that several nonsynonymous genetic variants in *GLP1R* alter the function of GLP-1 receptor and thereby affect the insulin secretion response to GLP-1 [89,90,91,92,93], most pharmacogenetic studies of GLP-1 receptor agonists to date have also been highly focused on *GLP1R* gene (Table 3).

### 3.1. GLP1R

Since Beinborn et al. [90] described the genetic link between *GLP1R* and responses to exendin-4 through in vitro experiments in 2005, it took 10 years for the clinical impact of *GLP1R* genetic polymorphisms in GLP-1 receptor agonist treatment to be first elucidated. Lin et al. [94] identified a total of 19 SNPs in the *GLP1R* gene with more than 20% allele frequency in 36 patients with T2D. Among these, one missense SNP (rs3765467 C > T) and another SNP (rs761386 C > T), which is in complete linkage disequilibrium (*r*^2^ = 1) with rs5875654 short tandem repeat with 2-base pair deletion polymorphism (8GA/7GA), were selected for the study. Both SNPs showed significant associations with changes in the standard deviation of plasma glucose (SDPG) levels between baseline and after treatment of exenatide 5 μg twice daily, in the presence of continuous subcutaneous insulin infusion (*p* = 0.041 for rs3765467; *p* = 0.019 for rs761386), with opposite effects on SDPG observed in rs3765467 (decreased) and rs761386 (increased), respectively. In addition, similar trends were exhibited even after multiple linear regression analyses were performed with the adjustment of covariates. SDPG is considered an independent risk factor for T2D complications because hypoglycemia from high glucose variability or fluctuations causes oxidative stress, endothelial dysfunction, and inflammatory reactions, which can lead to the development of vascular complications in T2D [102]. Results from the 75 g oral glucose tolerance test (OGTT) also showed that the T-allele in rs761386 is significantly associated with higher plasma glucose levels at 120 min after glucose intake (*p* = 0.032). However, this relationship became statistically insignificant after multiple linear regression analyses.

Subsequent research on the genetic association between *GLP1R* and GLP-1 receptor agonists mainly focused on their weight-lowering potential. de Luis et al. [95] investigated the effects of *GLP1R* rs6923761 variant on metabolic parameters and body weight secondary to liraglutide treatment in 90 T2D patients diagnosed with overweight (46 males and 44 females; mean BMI 33.7 ± 5.9 kg/m^2^). All participants were in the state of unstable blood glucose control with metformin monotherapy, and received a daily dose of 1.8 mg liraglutide subcutaneously for 14 weeks. Patients with *GLP1R* rs6923761 GA or AA genotypes exhibited significantly decreased waist circumference, waist-to-hip ratio and systolic blood pressure after liraglutide treatment, along with significant changes in BMI, body weight, and fat mass that were also observed in GG genotype patients. Furthermore, BMI, body weight, fat mass, and waist circumference before and after liraglutide treatment were significantly lower in the GA/AA genotype groups compared to patients with the GG genotype. Biochemical parameters that reflect the risk of cardiovascular complications between GG and GA/AA genotype were similar, with significant reductions in blood glucose, homeostasis model assessment for insulin resistance (HOMA-IR), and HbA1c levels after liraglutide administration. Only *GLP1R* rs6923761 genotype (GG vs. GA + AA) remained as an independent predictor for changes in body weight and fat mass in age- and sex-adjusted multivariate analyses.

Another pilot study by Jesnterle et al. [96] also evaluated the effects of *GLP1R* rs10305420 C > T and rs6923761 G > A variants on liraglutide-mediated weight loss in 57 obese women with polycystic ovary syndrome (PCOS). After a 12-week treatment of liraglutide (1.2 mg daily use), they were classified into two groups according to the clinical responses to liraglutide (20 strong responders and 37 poor responders), and baseline characteristics and *GLP1R* genotype frequency in each study group were compared. Both groups had significant reductions in body weight, BMI, waist circumference, fasting plasma glucose (FPG), and plasma glucose at 120 min after OGTT by liraglutide treatment. Furthermore, strong responders showed significantly decreased visceral adipose tissue area and HOMA-IR score after liraglutide administration, which was not observed in the poor responders. Frequency of rs10305420 CT or TT genotypes were significantly higher in poor responders compared with strong responders (64.9% vs. 35.0%; OR = 0.27; 95% CI = 0.09–0.85; *p* = 0.025), whereas the rs6923761 variant allele was associated with better responses to liraglutide (GA + AA genotype frequency in poor responders vs. strong responders: 43.2% vs. 70.0%; OR = 3.06; 95% CI = 0.96–9.74; *p* = 0.058). In haplotype analysis for evaluation of the combined effect of both SNPs, the C-A haplotype showed the best response to liraglutide compared with the reference C-G haplotype (the most common in this study) (OR = 3.85; 95% CI = 1.24–11.96; *p* = 0.020).

More recently, effects of genetic variants in *GLP1R* or *TCF7L2* in delayed gastric emptying and related weight reduction in obese patients with liraglutide or exenatide treatment were investigated [97]. This research was conducted as two different clinical trials: 5 μg exenatide (*n* = 10) or placebo (*n* = 10) was received twice daily for 30 days to obese individuals with rapid gastric emptying (defined as gastric emptying half-life (GE t_1/2_) less than 90 min) in the first study, and a daily dose of 3 mg liraglutide (*n* = 19; genetic evaluation performed in 18 subjects) or placebo (*n* = 21) was administered over 16 weeks in obese subjects with normal or rapid gastric emptying in the second study. Patients carrying *GLP1R* rs6923761 A-allele (GA or AA genotype) showed increased changes in GE t_1/2_ from baseline to 30 days (exenatide) or 5 weeks (liraglutide) after treatment compared with the GG genotype patients (117.9 ± 27.5 min vs. 98.5 ± 30.4 min in exenatide trial; 128.9 ± 38.3 min vs. 61.4 ± 21.4 min in liraglutide trial). Although these results were not statistically significant, the authors stated that a mean difference of approximately 65 min observed in liraglutide trial might be biologically relevant in terms of the association between gastric emptying and caloric intake. *GLP1R* rs6923761 SNP did not affect mean changes in body weight before and after GLP-1 receptor agonist treatment. Also, the *TCF7L2* rs7903146 variant was found to not be associated with the clinical efficacy of GLP-1 receptor agonists on gastric emptying and body weight reduction.

In a randomized, double-blind, parallel-group, single-center study for the efficacy and safety of dual therapy with dapagliflozin 10 mg and once-weekly 2 mg long-acting exenatide, pharmacogenetic analyses with several genes relevant to drug responses were performed in 40 obese patients without T2D [98]. However, all three selected *GLP1R* SNPs (rs10305420, rs6923761, and rs1042044) were not associated with changes in body weight after 24 or 28 weeks of concomitant treatment of dapagliflozin and exenatide.

### 3.2. Cannabinoid Type 1 Receptor (CNR1)

Cannabinoid type 1 receptor is widely distributed in various mammalian tissues including adipose tissue [103], and several previous studies have shown the association between the silent intragenic G1359A polymorphism (rs1049353) in *CNR1* and changes in body weight and metabolic status in obese subjects with hypocaloric diets [104,105,106]. As a follow-up study, de Luis et al. [99] investigated the effects of *CNR1* G1359A polymorphism (rs1049353) on weight loss and cardiovascular risk after a 14-week liraglutide treatment in 86 T2D patients with obesity (defined as BMI > 30 kg/m^2^). Patients were divided into two groups according to *CNR1* genotype, G1359G (GG; *n* = 45) and G1359A or A1359A (GA/AA; *n* = 35) groups. Patients in the GA/AA genotype group had significantly lower BMI, body weight, fat mass, and waist circumference before and after liraglutide treatment compared with GG genotype patients. Significant changes in plasma cholesterol levels after liraglutide treatment were observed in only GG genotype, whereas the 1359A allele was closely associated with improved insulin resistance, expressed as reduced HOMA-IR. Nevertheless, liraglutide administration resulted in similar improvements in most anthropometric characteristics (BMI, body weight, fat mass, waist circumference, waist-to-hip ratio, and systolic blood pressure), and basal glucose and HbA1c in both genotype groups.

### 3.3. Sortilin Related VPS10 Domain Containing Receptor 1 (SORCS1)

The relationship between *SORCS1* gene and T2D susceptibility has been reported by several previous studies [107,108,109]. Zhou et al. [100] assessed the influence of *SORCS1* rs1416406 G > A polymorphism on clinical efficacy of exenatide. A total of 101 Chinese T2D patients who participated in the CONFIDENCE study received 48-week exenatide treatment, and the relationship between rs1416406 SNP and glucose-lowering properties of exenatide was assessed by multiple linear regression analyses. Significant differences in HbA1c, FPG, 2-h PG, and β-cell function before and after exenatide treatment were observed in all three *SORCS1* rs1416406 genotype groups (GG, GA, and AA genotypes). However, with the exception of for proinsulin/insulin ratio (PI/I), changes in these parameters by exenatide between genotype groups did not reach statistical significance. *SORCS1* rs1416406 maintained a significant association with PI/I change (greater reduction in GG genotype) in multiple linear regression analyses adjustment of other co-variates.

### 3.4. WFS1 and TCF7L2

As aforementioned, Pereira et al. [98] performed pharmacogenetic analyses using seven selected SNPs in *GLP1R*, *TCF7L2*, *KCNQ1*, *WFS1*, and interleukin-6 receptor (*IL6R*) genes in 40 obese patients not diagnosed with T2D and demonstrated that *WFS1* rs10010131, a SNP which is known to be associated with impaired insulin secretion mediated by GLP-1 and increased T2D risk [110,111], was only significantly associated with body weight loss by combination therapy with dapagliflozin and once-weekly exenatide in the dominant (mean difference = −3.4 kg; 95% CI = −6.5 to −0.2; *p* = 0.0434) and additive models (mean difference = −2.4 kg; 95% CI = −4.5 to −0.3; *p* = 0.0337). Furthermore, the additive effects of *WFS1* rs10010131 retained significant associations with both kilogram- (*β* = −1.598; *p* = 0.0266) and percent change (*β* = −1.565; *p* = 0.0166) in body weight at 24 weeks after drug treatment after using multivariate generalized linear model analyses. Other genes were found to not be significantly associated with responses to dapagliflozin and exenatide dual therapy.

In contrast, the latest study conducted by Ferreira et al. [101] showed a significant relationship between the *TCF7L2* rs7903146 variant and glycemic responses to exenatide. Of the 162 T2D patients who were genotyped for *TCF7L2* rs7903146 SNP, a subgroup of 56 patients underwent a 500-kcal mixed-meal test with 8-week exenatide treatment and 46 patients completed the test (21 for CC genotype and 25 for CT/TT genotype). Plasma glucose, HbA1c, and body weight, and their changes before and after treatment were similar between the two genotype groups. Meanwhile, significantly higher basal insulin and proinsulin levels (both *p* < 0.05), and reductions in insulin (*p* < 0.05), proinsulin, and C-peptide (both *p* < 0.001) after exenatide treatment were observed in CT/TT genotype patients compared with the CC genotype group. The authors found the following: lower elevation in insulin levels after exenatide treatment in the rs7903146 CT/TT genotype group was attributed to secretion of a more efficient insulin, improved insulin sensitivity, or an increase in glucose uptake by the action of exenatide; and, due to the relationship between higher proinsulin level and impaired β-cell function and insulin resistance [112,113], a greater reduction in proinsulin indicated that use of GLP-1 receptor agonists may play a more important role in β-cell function in patients with CT/TT genotype. Indeed, significantly lower insulin resistance after exenatide treatment was observed in patients carrying the T-allele (*p* = 0.042), despite the fact that there was no remarkable difference in the estimated β-cell function before and after treatment between rs7903146 CC and CT/TT genotype groups.

## 4. SGLT2 Inhibitors

Since glucose reuptake kinetics in kidney were first demonstrated in the late 1930s [114], two main glucose transport systems in renal proximal tubules—SGLT1 and SGLT2—have been identified [115,116,117,118,119]. SGLT2, the most prevalent SGLT subtype at the early proximal tubule with low affinity and high capacity for glucose [119], accounts for more than 90% of renal glucose reabsorption in normoglycemic conditions. However, high blood glucose levels cause the growth of proximal tubules and SGLT2 expression, leading to increases in glucose reabsorption and the risk of undesirable diabetes [120,121,122]. Therefore, induction of glucosuria via SGLT2 inhibition has been considered a key mechanism of renal glucose homeostasis and a novel promising target for the treatment of T2D, and many efforts to develop new SGLT2 inhibitors from synthetic chemicals and naturally occurring constituents are still in progress [123,124,125].

The origin of currently available SGLT2 inhibitors is phlorizin, a dihydrochalcone isolated from the bark of apple trees [126]. Since showing that phlorizin inhibits renal glucose reabsorption and induces urinary glucose excretion [127], studies using diabetic animal models have demonstrated the association between phlorizin administration and reduced plasma glucose levels and improved insulin sensitivity [128,129,130]. More recently, phlorizin has shown nonselective SGLT inhibition with inhibitory constant (*Ki*) values of 151 and 18.6 nM toward human SGLT1 and SGLT2, respectively [131]. However, several drawbacks, including poor oral bioavailability, impaired glucose uptake in various tissues by the hydrolytic metabolite phloretin, and gastrointestinal adverse reactions via intestinal SGLT1 inhibition, have raised questions about clinical applicability of phlorizin as a novel antidiabetic agent [132].

Pharmaceutical companies have focused on the synthesis of phlorizin structure-based analogs that overcome these shortcomings with selectivity against SGLT2, resulting in the development of a phlorizin C-glucoside derivative dapagliflozin in 2008 [133]. Dapagliflozin exhibits more than 1200-fold higher potency against human SGLT2 compared with SGLT1, and its effects on T2D have been investigated in preclinical [133,134] and clinical practices [135,136,137,138,139,140,141,142]. Dapagliflozin was first approved by the European Medicines Agency (EMA) in 2012, and then the U.S FDA allowed the use of this medication for the treatment of T2D in 2014 [143]. Other new C-glucoside derivatives have been subsequently discovered following the emergence of dapagliflozin, and a total of seven SGLT2 inhibitors (canagliflozin, dapagliflozin, empagliflozin, ertugliflozin, ipragliflozin, luseogliflozin, and tofogliflozin) are now clinically available worldwide [144].

Since SGLT2 inhibitors are relatively recently marketed compared with other antidiabetic agents, the evaluation of efficacy and safety of these medications in various clinical settings has not yet been fully established. Nevertheless, SLGT2 inhibitors have shown significant decreases in blood glucose and HbA1c levels regardless of insulin amount or sensitivity with no weight gain or hypoglycemia, which are common side effects in conventional antidiabetic drugs [145,146]. Furthermore, several large-scale clinical outcome trials have demonstrated that SGLT2 inhibitors have therapeutic benefits in cardiovascular and renal systems [147,148,149,150,151,152]. With GLP-1 receptor agonists, the ADA-EASD consensus states that SGLT2 inhibitors are preferably considered in T2D patients with established ASCVD, heart failure, or CKD, and patients who need to minimize weight-related and excessive hypoglycemic problems (if the estimated glomerular filtration rate (eGFR) is adequate) [10]. Table 4 describes genes associated with SGLT2 inhibitor treatment and their clinical outcomes.

### 4.1. Uridine Diphosphate-Glucuronosyltransferase (UGT) Gene Family

Previous studies have reported that SGLT2 inhibitors are metabolized by several UGT isozymes [153,157,158,159], which are highly polymorphic and show large interindividual variabilities in glucuronidation rate and therapeutic responses of various drugs [160,161]. However, to date, there have been few studies on the pharmacogenetic association between UGT and canagliflozin. Francke et al. [153] investigated the drug metabolizing enzymes involved in the biotransformation of canagliflozin in vitro and the effects of genetic polymorphisms in these enzymes on canagliflozin PK in humans. Of the 12 recombinant human UGT isoforms, UGT1A9 and UGT2B4 were found to have a major role in the formation of two metabolites M7 and M5 from canagliflozin O-glucuronidation, respectively. A pooled analysis was performed in five clinical trials with healthy participants and two trials with T2D patients (total *n* = 134). They were all genotyped for UGT1A9*3 (rs72551330 T > C; p.Met33Thr) and UGT2B4*2 (rs1080755 A > G) and their pharmacokinetic parameters for canagliflozin and its metabolites were determined. Statistical significance was not calculated due to the small sample size, and UGT1A9*3 allele carriers (*n* = 4) showed 45% higher dose-normalized steady-state AUC (AUC_τ,ss_) of canagliflozin compared with UGT1A9*1/*1 subjects (*n* = 130) (10,379.4 ng·h/mL vs. 7152.7 ng·h/mL). Meanwhile, dose-normalized canagliflozin AUC_τ,ss_ between different *UGT2B4* genotypes (*n* = 83 for UGT2B4*1/*1; *n* = 45 for *1/*2; *n* = 6 for *2/*2) were similar (7079.5 ng·h/mL vs. 7376.9 ng·h/mL vs. 8385.1 ng·h/mL). When the data were confined to those who also determined metabolite PK, the gap in dose-normalized AUC_τ,ss_ according to *UGT1A9* genotype was even greater (54% difference, 6895.0 ng·h/mL in UGT1A9*1/*1 (*n* = 62); 10,613.3 ng·h/mL in UGT1A9*1/*3 (*n* = 3)). The metabolite/parent (M/P) ratio for M7 AUC_τ,ss_ was 36% lower, while M/P ratio for M5 AUC_τ,ss_ was 35% higher in UGT1A9*1/*3 subjects, indicating that blockade of M7 metabolism due to decreased UGT1A9 activity resulted in compensatory activation in another metabolic pathway into M5. Slightly higher dose-normalized AUC_τ,ss_ and lower M/P ratio for both metabolites were also observed in patients with UGT2B4*1/*2 or *2/*2 genotypes. Although the authors stated that the effects of UGT2B4*2 allele on canagliflozin PK are not negligible, clinical implications of *UGT2B4* genetic polymorphisms are considered to be minimal compared with *UGT1A9*.

Hoeben et al. [154] established population pharmacokinetic modeling of canagliflozin with data from 1616 healthy volunteers and T2D patients in 14 clinical trials. From the final population pharmacokinetic model with subjects whose *UGT1A9* genotypes were identified, UGT1A9*3 allele carriers (*n* = 21) showed higher median dose-normalized AUC values compared with subjects not carrying this allele (*n* = 700) (ratio = 1.26; 95% CI = 1.08–1.44). However, presence of the UGT1A9*3 allele did not significantly affect canagliflozin dosage adjustment, in addition to other covariates including sex, age, body weight, and renal function.

### 4.2. Solute Carrier Family 5 Member 2 (SLC5A2)

The *SLC5A2* gene is a member of the sodium/glucose cotransporter gene family, and is responsible for encoding SGLT2. The effects of genetic variants in *SLC5A2* on the development of familial renal glucosuria and related glycemic conditions have already been demonstrated [162,163]. Meanwhile, there has only been one study in which the effects of common SNPs in *SLC5A2* on risk factors for T2D and SGLT2 inhibitor treatment responses were investigated [155]. Two separate studies (cross-sectional study in 2229 individuals without diabetes; pharmacogenetic study in 979 patients from four placebo-controlled phase III clinical trials) were performed and six SNPs (rs9924771 G > A, rs11646054 G > C, rs3116149 G > A, rs9934336 G > A, rs3813008 G > A, and rs3116150 G > A) in *SLC5A2* with more than 5% minor allele frequencies were selected for genotyping. In cross-sectional study analyses, as the nominal effects, the rs3116150 A-allele showed increased FPG levels, glucose AUC during OGTT, and systolic blood pressure. Subjects in the pharmacogenetic study received 10 or 25 mg empagliflozin daily in their participating clinical trials, depending on the purpose of the research. After a 24-week treatment, rs3116150 and rs11646054 SNPs showed nominal association with changes in systolic blood pressure in the genotypic model (*p* = 0.0043 and *p* = 0.0350, respectively) and in the additive model (*p* = 0.0147 and *p* = 0.0142, respectively). Nominal associations between rs3116149 and changes in FPG (*p* = 0.0310) and systolic blood pressure (*p* = 0.0337) were also observed in the genotypic model. Other SNPs did not show significant and clinically relevant impacts on empagliflozin treatment responses. It was concluded that the effects of common *SLC5A2* genetic polymorphisms on clinical efficacy of empagliflozin were also negligible, as with *UGT* genes.

### 4.3. PNPLA3

As a part of clinical outcome trials on the efficacy of combination therapy with dapagliflozin and omega-3 carboxylic acids in T2D patients with NAFLD, Eriksson et al. [156] briefly demonstrated the association between *PNPLA3* rs738409 C > G genotype and therapeutic responses in different treatment groups. In the combined treatment arm, reduction in PDFF levels was significantly higher in the CG + GG genotype group (relative change = −25.4%; 95% CI = −27.3 to −19.0%) compared with the CC genotype group (relative change = −16.1%; 95% CI = −20.5 to −11.6%) (*p* < 0.01). On the other hand, dapagliflozin monotherapy showed completely opposite results between the two genotype groups (CG + GG: relative change = 7.0%; 95% CI = −2.2 to 11.3%, CC: relative change = −22.0%; 95% CI = −26.8 to −19.2%, *p* < 0.01).

## 5. Future Perspectives and Challenges

Demand for personalized therapy based on the individual traits of patients is constantly increasing, and precision medicine strategies to prevent and treat various medical conditions are being established in several developed countries. Because chronic diseases, such as cancer, hypertension, dyslipidemia, asthma, and diabetes, have high incidence rates and are associated with the risk of complications, clinical impacts of therapeutic approaches through precision medicine would be of a greater magnitude.

A variety of medications with different mechanisms of action have been developed for glycemic control in T2D patients, but the use of most conventional drugs except metformin has been steadily decreasing mainly due to the risk of hypoglycemia and negative effects on cardiovascular systems. Meanwhile, recently developed antidiabetic agents including DPP-4 inhibitors, GLP-1 receptor agonists and SGLT2 inhibitors are relatively beneficial for these problems. From extensive clinical pharmacogenetic studies, some noteworthy associations between drug class and genes were identified: DPP-4 inhibitors and *DPP4*, *GLP1R*, *TCF7L2*, *PNPLA3*, *CDKAL1*, *KCNJ11*, *KCNQ1*, and *PRKD1*; GLP-1 receptor agonists and *GLP1R*, *CNR1*, *SORCS1*, *WFS1*, and *TCF7L2*; and SGLT2 inhibitors and *UGT1A9*, *SLC5A2*, and *PNPLA3* (Table 2, Table 3 and Table 4). Since most studies have been conducted based on very limited study designs and sample size and thus somewhat inconsistent outcomes have been shown, additional large-scale clinical trials and GWAS approaches are needed to clarify the clinical implications of these genes and to identify novel genetic biomarkers in the prevention and treatment of T2D and its complications. More recently, “multiomic” applications integrating genetic, metabolomic and/or proteomic data are emerging in the field of disease risk prediction model, providing information on the mutual impacts of genetic variations and metabolic differences for the early diagnosis, treatment, and prevention of various complex diseases including T2D [164,165].

Genetic polymorphisms in drug-metabolizing enzymes and drug transporters and their influence on drug PK and/or PD are the most studied and are well-established in the field of pharmacogenetics and pharmacogenomics. Many studies have provided information on proteins involved in the disposition of GLP-1-based medications and SGLT2 inhibitors. For example, sitagliptin undergoes oxidative metabolism via CYP3A4 and CYP2C8 [166], and is transported by organic anion transporter 3 (OAT3), organic anion transporting polypeptide 4C1 (OATP4C1), and P-gp [42]. The main drug-metabolizing enzymes responsible for the metabolism of saxagliptin are CYP3A4 and CYP3A5 [167], and linagliptin is a substrate for OCT2 and P-gp transporters [168]. Similar to canagliflozin, dapagliflozin is hepatically and renally metabolized by UGT1A9 [169]. In a recent preclinical study [170], OAT3-mediated glucosuric effect of empagliflozin has been reported. Although the effects of single enzyme or transporter on drug PK and PD are small and previous clinical pharmacogenetic studies have also not shown clinically significant relationships [41,153,154], the combined effects of multiple gene and proteins or interactions with other factors affecting drug disposition (e.g., sex, age, organ function, concomitant drugs, comorbid diseases, and social and environmental factors) should not be overlooked.

Recent clinical guidelines for the management of hyperglycemia in T2D recommends early combination therapy for patients with insufficient therapeutic benefits from metformin alone and greater reduction in HbA1c is required [10,171], and the clinical efficacy and safety of initial combined regimen with a DPP-4 inhibitor, GLP-1 receptor agonist or SGLT2 inhibitor and metformin, or with two newer antidiabetic medications in T2D treatment are still being evaluated [172,173,174]. Therefore, future pharmacogenetic studies should include analyses of complex effects of multiple genes associated with drugs used in combination therapy.

## 6. Conclusions

In the era of precision medicine, accurate disease diagnoses, and selection of the most optimal therapeutic strategy for individualized treatment will be realized, and early detection and prevention of diseases will also feasible. Pharmacogenetic-based tailored medicine and targeted therapy will play key roles in precision medicine, and will act as cornerstones for its establishment and advancement in clinical practices.

We presented pharmacogenetic biomarkers in clinical responses to newer second-line antidiabetic medications in this review, as a useful tool for precision medicine in T2D. Functional variations in these genes significantly affected drug-related glycemic control, adverse reactions and risk of diabetic complications. More comprehensive pharmacogenetic researches reflecting different and complex clinical settings will allow us to confirm the combined effects of multiple genes and discover novel biomarkers or therapeutic targets for the management of T2D.

## Figures and Tables

**Table 1:** List of drug classes available for the treatment of type 2 diabetes (T2D).

Drug Class	Examples ^1^ (Currently Marketed as a Single Active Ingredient)
**Oral**	
α-Glucosidase inhibitors	Acarbose, miglitol
Biguanides	Metformin
Bile acid sequestrants	Colesevelam
DPP-4 inhibitors	Alogliptin, linagliptin, saxagliptin, sitagliptin, vildagliptin
Dopamine-2 agonists	Bromocriptine
Meglitinides	Nateglinide, repaglinide
SGLT2 inhibitors	Canagliflozin, dapagliflozin, empagliflozin, ertugliflozin
Sulfonylureas	Chlorpropamide, gliclazide, glimepiride, glipizide, glyburide (glibenclamide), tolazamide, tolbutamide
Thiazolidinediones	Pioglitazone, rosiglitazone
**Injectable**	
Amylin analogs	Pramlintide
GLP-1 receptor agonists	Albiglutide, dulaglutide, exenatide, liraglutide, lixisenatide, semaglutide
Insulin and its analogs	Insulin aspart, insulin degludec, insulin detemir, insulin glargine, insulin glulisine, insulin human, insulin lispro, human NPH (neutral protamine Hagedorn), human regular

^1^ approved by the U.S FDA and/or European Medicines Agency (EMA) (Ref. [10,15]). DPP-4, dipeptidyl peptidase-4; GLP-1, glucagon-like peptide-1; SGLT2, sodium-glucose cotransporter-2.

**Table 2:** Genes associated with responses to DPP-4 inhibitors.

Gene	Study Population	Dosage	Genetic Variant(s)	Clinical Outcome(s)	Ref.
*DPP4*	27 T2D patients with hypertension and 38 healthy controls	Sitagliptin 100 mg/day or a single dose of 200 mg	rs2909451 rs759717	Increased DPP-4 activity during sitagliptin treatment in rs2909451 TT genotype and rs759717 CC genotype; rs2909451 genotype was only considered a predictive factor for DPP-4 activity	[32]
*GLP1R*	246 Korean patients with T2D	Various (dosage not provided)	rs3765467	Higher reduction in HbA1c and higher DPP-4 inhibitor responder proportion observed in GA/AA genotype	[33]
	140 white patients treated for T2D in outpatient clinics	Sitagliptin or vildagliptin 100 mg/day	rs6923761 (p.Gly168Ser)	Lower reduction in HbA1c in Ser/Ser genotype	[34]
*TCF7L2*	693 T2D patients from four phase III clinical trials	Linagliptin 5 mg/day	rs7903146	Lower decreases in HbA1c and 2-h PG levels from baseline in TT genotype	[35]
*PNPLA3*	41 patients with T2D and NAFLD	Alogliptin 25 mg/day	rs738409 (p.Ile148Met)	Positive correlation between improvement in HbA1c and changes in liver aminotransferase levels in CG/GG genotype; higher reductions in total cholesterol, hyaluronic acid, and triglyceride in CG/GG genotype	[36]
*CDKAL1*	512 T2D patients receiving DPP-4 inhibitor treatment	Various (dosage not provided)	rs7754840 rs7756992	Higher reduction in HbA1c in rs7754840 CC genotype and rs7756992 GG genotype	[37]
*KCNJ11*	662 subjects with T2D (331 receiving DPP-4 inhibitor and 331 receiving other medications)	Sitagliptin 100 mg/day; vildagliptin 50–200 mg/day; linagliptin 5 mg/day	rs2285676	Strong association between poor DPP-4 inhibitor efficacy and T-allele (OR = 1.479; 95% CI = 0.753–1.403), as a genetic predictor of DPP-4 inhibitor treatment response	[38]
*KCNQ1*	137 European patients from five outpatient clinics treated for T2D	Sitagliptin or vildagliptin 100 mg/day	rs163184	Less reduction in HbA1c in G-allele carriers	[39]
*PRKD1*	88 resistant and 83 sensitive responders to T2D treatment	Various (dosage not provided)	rs57803087	Strong association with DPP-4 inhibitor response and rs57803087 SNP from GWAS and replication study	[40]

2-h PG, 2-h postprandial plasma glucose; DPP-4, dipeptidyl peptidase-4; GWAS, genome-wide association study; NAFLD, non-alcoholic fatty liver disease; SNP, single-nucleotide polymorphism; T2D, Type 2 diabetes.

**Table 3:** Genes associated with responses to GLP-1 receptor agonists.

Gene	Study Population	Dosage	Genetic Variant(s)	Clinical Outcome(s)	Ref.
*GLP1R*	36 patients with T2D	Exenatide 5 μg twice daily	rs3765467 rs761386 (in complete LD (*r*^2^ = 1) with rs5875654)	Decreased (rs3765467 CT/TT) or increased (rs761386 CT/TT) SDPG levels; higher 2-h PG after 75 g OGTT in rs761386 CT/TT genotype	[94]
	90 patients with T2D and overweight (BMI > 25 kg/m^2^)	Liraglutide 1.8 mg/day	rs6923761	Decreased waist circumference, waist-to-hip ratio and systolic blood pressure in GA/AA genotype, as an independent predictor for weight and fat mass reduction	[95]
	20 strong responder and 37 poor responder obese women with PCOS	Liraglutide 1.2 mg/day	rs10305420 rs6923761	Higher rs10305420 T-allele in poor responders; higher rs6923761 A-allele in strong responders; the best response to liraglutide observed in combined C-A haplotype (OR = 3.85; 95% CI = 1.24–11.96)	[96]
	20 obese individuals with rapid gastric emptying (exenatide) and 40 obese individuals with normal or rapid gastric emptying (liraglutide)	Exenatide 5 μg twice daily; liraglutide 3 mg/day	rs6923761	Prolonged gastric emptying half-life in GA/AA genotype (more remarkable in liraglutide treatment); no effect on body weight	[97]
*WFS1*	40 obese patient without T2D	Long-acting exenatide 2 mg/week (plus dapagliflozin 10 mg)	rs10010131	Higher body weight loss in A-allele carriers	[98]
*CNR1*	86 patients with T2D and obesity (BMI > 30 kg/m^2^)	Liraglutide 1.8 mg/day	rs1049353 (G1359A)	Decreased total cholesterol and LDL-cholesterol in GG genotype; improved HOMA-IR in GA/AA genotype	[99]
*SORCS1*	101 newly diagnosed T2D patients from CONFIDENCE study	Exenatide 5 μg twice daily (week 1–4) 10 μg twice daily (week 5–48)	rs1416406	Higher reduction in proinsulin/insulin ratio in GG genotype	[100]
*TCF7L2*	46 patients with T2D completed a 500-kcal mixed-meal test	Exenatide 5 μg twice daily (week 1–4) 10 μg twice daily (week 5–8)	rs7903146	Higher basal insulin and proinsulin, and reductions in insulin, proinsulin and C-peptide in CT/TT genotype	[101]

2-h PG, 2-h postprandial plasma glucose; BMI, body mass index; HOMA-IR, homeostasis model assessment for insulin resistance; LD, linkage disequilibrium; OGTT, oral glucose tolerance test; PCOS, polycystic ovary syndrome; SDPG, standard deviation of plasma glucose; T2D, Type 2 diabetes.

**Table 4:** Genes associated with responses to sodium-glucose cotransporter-2 (SGLT2) inhibitors.

Gene	Study Population	Dosage	Genetic Variant(s)	Clinical Outcome(s)	Ref.
*UGT1A9*	134 healthy participants and T2D patients from 7 clinical trials	Canagliflozin 50–300 mg/day	UGT1A9*3 (rs72551330; p.Met33Thr)	Higher dose-normalized steady-state AUC (AUC_τ,ss_) for canagliflozin and M5, M/P ratio for M5 AUC_τ,ss_; lower AUC_τ,ss_ for M7, M/P ratio for M7 AUC_τ,ss_ (UGT1A9*3 carriers)	[153]
	1616 healthy volunteers and T2D patients from 14 clinical trials	Canagliflozin 50–1600 mg/day	UGT1A9*3 (rs72551330; p.Met33Thr)	Higher median dose-normalized canagliflozin AUC in UGT1A9*3 carriers (ratio = 1.26; 95% CI = 1.08–1.44)	[154]
*SLC5A2*	979 patients from 4 phase III clinical trials	Empagliflozin 10 or 25 mg/day	rs3116650 rs3116149 rs11646054	Increased systolic blood pressure by rs3116650 A-allele, rs3116149 A-allele and rs11646054 C-allele; decreased FPG levels in rs3116149 AA genotype	[155]
*PNPLA3*	20 dapagliflozin alone and 20 dapagliflozin plus omega-3 carboxylic acids treated subjects with T2D and NAFLD	Dapagliflozin 10 mg/day	rs738409 (p.Ile148Met)	Lower reduction in liver PDFF in dapagliflozin alone treatment group; higher reduction in liver PDFF in dapagliflozin plus omega-3 carboxylic acids treatment group (CG/GG genotype)	[156]

AUC, area under the plasma concentration–time curve; FPG, fasting plasma glucose; M/P ratio, metabolite/parent ratio; NAFLD, non-alcoholic fatty liver disease; PDFF, proton density fat fraction; SNP, single-nucleotide polymorphism; T2D, Type 2 diabetes.
